# Sphericity Control of UO_2_ Fuel Kernels Through Gelling Media Coupling with Multi-Field Washing

**DOI:** 10.3390/ma19081484

**Published:** 2026-04-08

**Authors:** Laiyao Geng, Hui Jing, Yanli Zhao, Jia Li, Xiaolong Liu, Yongjun Jiao, Yong Xin, Yuanming Li, Hailong Qin, Xin Li, Shan Guo

**Affiliations:** 1 State Key Laboratory of Advanced Nuclear Energy Technology, Nuclear Power Institute of China, Chengdu 610213, China; genglaiyao@163.com (L.G.); lijia@npic.ac.cn (J.L.); liuxiaolong@npic.ac.cn (X.L.); xinyong@npic.ac.cn (Y.X.); liyuanming@npic.ac.cn (Y.L.); qinhailong@npic.ac.cn (H.Q.); lixin@npic.ac.cn (X.L.); 13997976561@163.com (S.G.); 2 The State Key Laboratory of Polymer Materials Engineering, Polymer Research Institute, Sichuan University, Chengdu 610065, China; 2022323095102@stu.scu.edu.cn

**Keywords:** sol–gel, UO_2_ sphericity, silicone oil, static washing

## Abstract

Nuclear energy has emerged as a crucial technological solution for ensuring energy security and achieving carbon neutrality goals, given its ultra-high energy density and near-zero carbon emissions against the backdrop of rapid socioeconomic development, increasing energy demands, and accelerated global transition toward low-carbon energy structures. As the core component for energy conversion in nuclear reactors, fuel elements critically determine reactor efficiency and safety performance, with the fission product retention capability of silicon carbide layers in multilayer-coated fuel particles having been thoroughly validated through high-temperature gas-cooled reactor irradiation tests. The precise sphericity control of large-sized UO_2_ fuel kernels represents a fundamental requirement for enhancing tristructural isotropic (TRISO) fuel particle performance and advancing Generation IV nuclear power plant development. This study presents a sphericity control strategy based on sol–gel processing that synergistically integrates physicochemical regulation of gelling media with multi-field washing flow field optimization. By implementing silicone oil-mediated interfacial tension gradient control, we effectively suppressed gel sphere destabilization while developing an innovative three-phase sequential washing technique involving kerosene washing, anhydrous ethanol interfacial transition, and ammonia solution replacement, which significantly enhanced mass transfer diffusion in stagnant liquid films and revolutionized fuel microsphere washing technology with improved efficiency and quality. Experimental results demonstrate that this integrated approach increases kernel sphericity qualification to 99.8%, reduces washing solution consumption by 79%, and achieves an average sphericity of 1.03. The research establishes a coupling mechanism between gelling media and multi-field washing processes, elucidating the synergistic effect between interfacial tension regulation and washing optimization, thereby providing both theoretical foundations and engineering application basis for the precision manufacturing of high-performance nuclear fuels.

## 1. Introduction

Nuclear energy has emerged as a pivotal low-carbon energy source for achieving global carbon neutrality goals [1,2], with the International Atomic Energy Agency projecting its installed capacity to increase by over 40% by 2050 [3,4]. Among advanced nuclear technologies [5,6,7], high-temperature gas-cooled reactors (HTGRs) have become a key pathway for fourth-generation nuclear systems [8,9,10], due to their inherent safety and broad potential for high-temperature applications [11,12,13,14,15]. These reactors utilize tristructural isotropic (TRISO) fuel particles [16,17,18,19,20,21] whose performance is highly sensitive to the geometric perfection of uranium dioxide (UO_2_) fuel pellets [22,23,24]. Numerous studies have conducted analytical evaluations of TRISO fuel performance [25,26,27]. For instance, Hales et al. investigated the effects of geometric deformation on TRISO particle performance under irradiation and accident conditions [26,28], revealing that reduced sphericity significantly alters thermal conductivity and stress distribution patterns, induces local stress concentration, and elevates the probability of interlayer rupture. For particles with defects such as thinning of the silicon carbide layer, such deformation further complicates the diffusion pathways of fission products (e.g., curium), increasing their release by approximately 60% compared to ideal spherical particles. Additionally, deformation in high-temperature environments compromises the SiC layer’s containment capability for fission products [22]. Experimental data from Miller et al. in the NPR project indicated that less than 5% of particles exhibit sphericity exceeding 1.12 within a 95% confidence interval. Simulation analyses of non-spherical TRISO-coated fuel particles using PARFUME demonstrated that non-spherical effects significantly degrade performance under accident conditions, recommending that sphericity be controlled within 1.1 to ensure safety [29].

Since the last century, the sol–gel method has remained the mainstream process for manufacturing nuclear fuel microspheres [30,31,32], owing to its unique advantages in ensuring compositional homogeneity [33,34], enabling low-temperature synthesis, and precisely controlling particle morphology [35,36]. Widely adopted for fabricating high-performance fuels like tristructural isotropic particles in high-temperature gas-cooled reactors [37,38], it outperforms traditional powder metallurgy by avoiding high-energy milling and reducing impurity risks [39,40]. Ganatra et al. successfully prepared fuel microspheres (100 ± 20 μm) via the internal gelation method, involving three carbon tetrachloride washes and six ammonia washes. The resulting microspheres featured a smooth surface, were crack-free, and achieved a density of 10.85 g/cm^3^ [41]. Guo et al. employed a full gelation process—encompassing solution preparation, droplet formation and gelation, washing, and aging—to produce 500 ± 50 μm fuel microspheres with sphericity superior to 1.2, though no further investigation into sphericity control was pursued [42]. Xu et al., using zirconia (ZrO_2_) microspheres as surrogates for UO_2_ nuclear fuel, fabricated crack-free, smooth-surfaced, and dense ZrO_2_ ceramic microspheres via an optimized internal gelation and washing process, achieving a density of 99% of the theoretical value [43]. Chernikov et al. fabricated UO_2_ fuel spheres with a sphericity of 1.05 in response to the relevant requirements of high-temperature gas-cooled reactors [44]. Brykala et al. employed the sol–gel method to prepare small ThO_2_ microspheres, achieving a controlled sphericity of 1.10 [45]. Collectively, these achievements reveal a fundamental trilemma in large-sized pellet production: the trade-off among sphericity precision, impurity removal, and manufacturing yield [20,46,47,48].

In this study, through innovative process integration, we propose a systematic solution: during the gelation stage, a novel dimethyl silicone oil composite medium is introduced to effectively suppress phase separation disturbances; the washing process is reconfigured into a three-stage sequence comprising static permeation, dynamic flushing, and chemical dissociation. Systematic evaluation demonstrates that the alternating static–dynamic washing strategy achieves unprecedented performance metrics: a 99.8% sphericity qualification rate, a 33% reduction in washing liquid consumption, and an average sphericity of 1.03. These breakthroughs open new avenues for the industrial-scale production of TRISO fuel while deepening fundamental understanding of interfacial phenomena in nuclear fuel manufacturing. This method distinguishes itself significantly from conventional approaches by enabling simultaneous improvements in product quality, process efficiency, and economic feasibility, thereby providing critical support for the development of advanced nuclear energy systems.

## 2. Materials and Methods

### 2.1. Materials

Silicone oils with viscosities of 350 mPa·s and 100 mPa·s were purchased from Dow Corning in the Midland, MI, USA, while hexamethylenetetramine, urea, ethanol, isooctanol, nitric acid, and kerosene of analytical grade were obtained from Sinopharm Chemical Reagent Co., Ltd. (Shanghai, China), with deionized water demonstrating a resistivity of 18.0 MΩ·cm and uranyl nitrate solution being prepared through laboratory synthesis.

### 2.2. Sample Preparation

**Figure 1:**
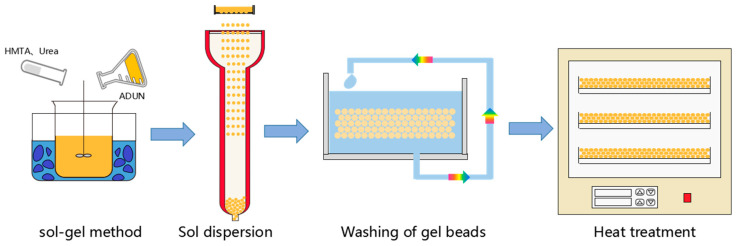
Process flowchart for the fabrication of large diameter UO_2_ microspheres.

In this experiment, uranium oxide gel spheres were prepared from acid-deficient uranyl nitrate (ADUN) solution using the internal gelation process. The ADUN solution was mixed with gelling agents hexamethylenetetramine and urea at temperatures at or below 5 °C to form a sol, which was then extruded through precision nozzles into a heated silicone oil column serving as the gelation bath. Within the hot silicone oil medium, internal hydrolysis and polycondensation reactions occurred in the sol droplets, causing uranium ion precipitation and formation of spherical wet gel spheres with three-dimensional network structures. The obtained wet gel spheres underwent multistage washing to completely remove residual silicone oil, unreacted reagents including HMTA and urea, and byproduct ions such as nitrate and ammonium through an alternating static and dynamic washing process using a specific solvent sequence of kerosene, anhydrous ethanol, and dilute ammonia solution. After washing, the wet gel spheres were dried to remove most water content before being calcined in a high-temperature furnace to decompose residual organic compounds and convert uranium oxides from UO_3_ to the desired UO_2_ form, ultimately producing dense uranium oxide calcined spheres with excellent sphericity, with the complete process flow illustrated in Figure 1.

#### 2.2.1. Preparation of Gel Spheres

In this experiment, isooctanol was initially employed as the gelling medium, which was subsequently replaced by dimethyl silicone oil, with the medium temperature stably maintained at 70 ± 1 °C via a constant-temperature water bath. The sol was dispersed into droplets by a vibrating nozzle under a carrier flow rate of 0.5 L/min of the gelling medium, with key dispersion parameters set as follows: vibration frequency of 80–110 Hz, carrier gas pressure of 0.03 MPa, and sol flow rate of 30 mL/min. Under these conditions, the formed sol droplets entered a gelling column filled with hot isooctanol/silicone oil mixture, where an internal gelation reaction occurred during natural sedimentation, gradually solidifying into spherical wet hydrogel spheres (as shown in Figure 1). The resulting hydrogel spheres then flowed into a bottom-connected aging tank, and were aged statically for 20 min at a constant temperature of 70 °C to reinforce their network structure. After aging, the hydrogel spheres were discharged and transferred to the subsequent washing process.

Throughout the experiment, the medium composition and temperature field were regulated to ensure uniform heating of sol droplets within the gelling column and complete phase transition and solidification. The parameter combination of the vibrating nozzle (frequency: 80–110 Hz, carrier gas pressure: 0.03 MPa) effectively suppressed droplet coalescence, ensuring the formation of monodisperse spherical particles, while high-temperature static aging in the aging stage promoted further cross-linking of sol particles, enhancing the mechanical stability of the gel skeleton. These process parameters provide well-uniform precursor samples for subsequent material property characterization, featuring strong reproducibility of operational details and suitability for large-scale preparation of spherical functional gel materials.

#### 2.2.2. Washing Process of Gel Spheres

This experiment employed a multistage washing process using analytical-grade kerosene, anhydrous ethanol, and 0.75 mol/L ammonia solution as cleaning agents, with different washing method combinations to ensure effective impurity removal. The dynamic washing followed a programmed procedure: two cycles of kerosene washing at 5 min per cycle, followed by two cycles of anhydrous ethanol washing at 5 min per cycle, and concluding with four cycles of ammonia solution washing at 30 min per cycle. Building upon this foundation, the introduction of static washing and hybrid dynamic–static washing modes significantly enhanced washing efficiency. Static washing prolonged the contact time between cleaning agents and materials to 30 min for kerosene, 20 min for ethanol, and 60 min for ammonia, thereby intensifying impurity diffusion. The hybrid dynamic–static washing alternately applies short-duration dynamic washing to facilitate cleaning agent renewal and extended static soaking to promote deep purification, with the total washing duration extended to twice that of the basic dynamic washing. Experimental results demonstrated that this composite washing approach effectively removed impurities such as nitrate ions, reducing residual levels below 50 ppm while maintaining the structural integrity of the gel spheres. The improved washing performance primarily resulted from three factors: first, the synergistic decontamination effect of multiple cleaning agents; second, the complementary interaction between dynamic flushing and static permeation; and third, the optimized washing durations tailored for different impurities.

## 3. Results and Discussion

### 3.1. Influence of Gelling Medium on the Sphericity of Gel Spheres

The sphericity of gel spheres serves as a critical determinant for the quality of multilayer-coated fuel kernels. This study revealed that the sphericity of sol droplets after gel formation directly influences the morphological characteristics of the final UO_2_ fuel kernels. Since the geometric morphology of gel spheres remains essentially unchanged during subsequent washing, drying–calcination, and reduction–sintering processes, controlling the initial sphericity at the gelation stage becomes paramount. Experimental results demonstrated that the selection of gelling medium constitutes the decisive factor affecting sphericity: on one hand, appropriate medium viscosity, exemplified by dimethyl silicone oil, effectively maintains droplet stability during formation; on the other hand, the surface tension properties of the medium significantly influence droplet deformation behavior. By optimizing the physical parameters of the gelling medium, sol droplets can achieve superior sphericity during the formation stage, thereby establishing a foundation for the subsequent production of high-sphericity UO_2_ fuel kernels. This finding provides crucial theoretical guidance for optimizing the manufacturing process of multilayer-coated fuels.

#### 3.1.1. Effect of Isooctanol Gelling Medium Temperature on Sphericity

**Figure 2:**
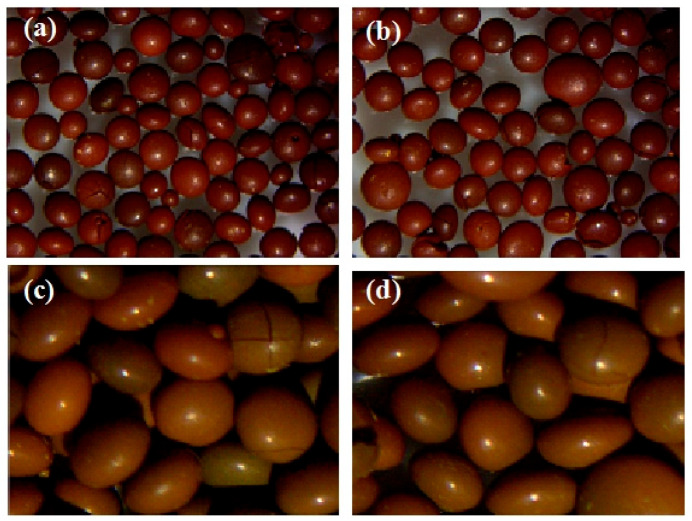
Morphology of dried and calcined spheres at different gelling temperatures: (**a**) 70 °C, (**b**) 75 °C, (**c**) 80 °C, (**d**) 85 °C.

In the preparation of uranium dioxide microspheres via the sol–gel method, isooctanol has conventionally served as the dispersion medium for small-diameter microspheres due to its density of 0.835 g/mL and viscosity of 9.8 mPa·s at 20 °C. For large-diameter gel sphere fabrication, this study proposed a theoretical hypothesis based on Stokes’ law that increasing gelling medium density could effectively reduce sol droplet sedimentation velocity in the gelation column, thereby minimizing deformation risks. However, experimental results (Figure 2) demonstrated significant droplet deformation within the predetermined optimization temperature range of 65–70 °C in isooctanol medium, with sphericity indices D_L_/D_S_ consistently exceeding 1.3, confirming that density adjustment alone cannot ensure spherical stability for large droplets. To investigate temperature parameter regulation potential, a dual-effect model of temperature influence was developed: at lower temperatures, gelation rate limitations from reaction kinetics resulted in insufficient gel sphere structural strength, prone to deformation from inter-particle compression, while temperature elevation accelerated solidification but concurrently reduced medium viscosity, diminishing its deformation resistance capability. Systematic experiments across 70–85 °C revealed progressive sphericity deterioration, with DL/DS increasing from 1.39 at 70 °C to 1.50 at 85 °C (Table 1), showing a linear regression slope of 0.036 per degree Celsius and a coefficient of determination of 0.92. Microscopic morphology analysis further confirmed elliptical morphology across all temperature points, with enhanced flattening trends at elevated temperatures, exemplified by D_S_ reduction to 1.6 mm at 85 °C. This phenomenon contradicted initial theoretical predictions. Mechanistic analysis indicated that high-temperature-induced viscosity reduction intensified internal flow field disturbances, triggering Rayleigh–Plateau interfacial instability that caused persistent droplet deformation before solidification. Consequently, although higher temperatures shortened gelation time, rheological property degradation exacerbated sphericity deterioration. These findings reveal inherent limitations of isooctanol for large-diameter high-sphericity microsphere production—its narrow property modulation window cannot balance solidification rate and interfacial stability, making single-parameter temperature optimization insufficient for sphericity improvement, thereby providing theoretical justification for alternative high-viscosity media such as silicone oil.

#### 3.1.2. Influence of Silicone Oil as the Gelling Medium on the Sphericity of Gel Spheres

Dimethyl silicone oil, also known as methyl silicone oil, is a colorless, transparent, viscous liquid and a high-molecular polymer with the following molecular formula: (CH_3_)_3_SiO[(CH_3_)_2_SiO]_n_Si(CH_3_)_3_. At room temperature, its density is 0.963 g/mL, and it exhibits excellent thermal stability over a wide temperature range, high light transmittance, and superior chemical inertness. It has low volatility, a high flash point, and a small viscosity–temperature coefficient. Its kinematic viscosity increases with molecular weight. Therefore, considering both the need to increase the density and viscosity of the dispersing medium, dimethyl silicone oil is an ideal choice for this purpose, enabling effective control of sol droplet sphericity.

Analysis of the motion of sol droplets in organic dispersing media reveals that the shape of larger droplets during dispersion is determined by the balance between forces promoting deformation and those resisting it. The cross-sectional area A of a deformed droplet can be expressed as follows:

(1) $$A=\frac{V}{h}\approx \frac{\varrho_{c}U^{2}V}{2\sigma }$$

ρc—medium density; σ—medium surface tension; U—droplet velocity; V—droplet volume.

Combining this with the equation of motion for deformed droplets in a gravitational field, the terminal velocity of the droplet can be estimated as follows:

U ≈ (4σρg/C_d_ρ_c_^2^)1/4 (2)

C_d_—drag coefficient of the dispersing medium.

The above equation shows that the terminal velocity U of the droplet is inversely proportional to the square root of the dispersing medium’s density and directly proportional to the fourth root of surface tension, indicating that as droplet velocity increases, the cross-sectional area expands, leading to deformation. Based on this analysis, to control the sphericity of gel spheres, the settling velocity U of sol droplets in the dispersing medium must be minimized. Force analysis of sol droplets in the dispersing medium confirms that increasing either the density or viscosity of the medium effectively reduces U. Density measurements show that the density of isooctanol is 0.832 g/mL and that of silicone oil is 0.963 g/mL. Additionally, the viscosity of silicone oil can be regulated by adjusting its molecular weight, thereby affecting the cross-sectional area of the deformed droplet; theoretical calculations further confirm that when the viscosity is optimized to 215 mPa·s, excellent sphericity can be achieved.

**Figure 3:**
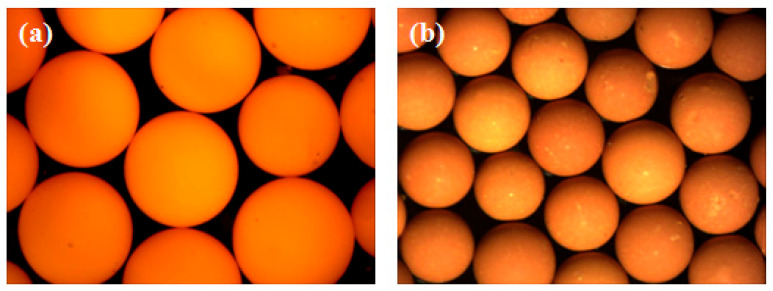
(**a**) Morphology of gel microspheres. (**b**) Morphology of calcined microspheres.

In this experiment, two types of silicone oil with viscosities of 100 mPa·s and 350 mPa·s were mixed at a volume ratio of 3:4, yielding a blended viscosity of 215 mPa·s at room temperature. Under experimental conditions of 70 °C dispersing medium temperature, 80–110 Hz vibration frequency, 30 mL/min sol flow rate, and 0.03 MPa dispersing pressure, the morphology of the resulting gel and dried-calcined spheres is shown in Figure 3. Microstructural analysis reveals that both gel and calcined spheres exhibit near-perfect spherical shapes, attributed to the optimized rheological properties of silicone oil: its higher density significantly reduces droplet settling velocity, while its high viscosity effectively suppresses Rayleigh–Plateau interfacial instability, preserving droplet shape prior to solidification. In contrast to the prevalent ellipsoidal deformation observed in isooctanol media, the silicone oil system maintains a sphericity (D_L_/D_S_) below 1.02 under identical temperature conditions, demonstrating its ability to overcome deformation limitations in large-diameter microsphere fabrication through synergistic control of density and viscosity parameters.

### 3.2. Influence of Washing Methods on the Sphericity of Gel Spheres

In the manufacturing process of uranium dioxide gel microspheres, the washing procedure serves to effectively remove surface residual dimethyl silicone oil and internal soluble impurities, including nitrate ions, formaldehyde, and ammonia, with nitrate presence confirmed as the primary cause of microsphere cracking during heat treatment. Since nitrate removal requires diffusion mass transfer into the washing solution, complete elimination of surface gelling media becomes a prerequisite for impurity extraction. This study employed dimethyl silicone oil as the dispersion medium, whose hydrophobic nature necessitated the use of low-boiling-point organic solvents such as kerosene or petroleum ether as primary cleaning agents, followed by ammonia solution for ionic impurity removal. Critical findings revealed that different washing modes significantly influenced microsphere surface morphology: static washing reduced mechanical damage but demonstrated low impurity removal efficiency; dynamic washing enhanced mass transfer efficiency yet frequently induced particle collision deformation; and a hybrid static–dynamic approach optimized washing cycles and intensity to maintain high mass transfer efficiency while effectively controlling mechanical stress. Surface quality analysis demonstrated that improved smoothness directly enhanced microsphere aspect ratios, with D_L_/D_S_ values measuring 1.32 ± 0.08 for dynamic mode, 1.03 ± 0.01 for static mode, and decreasing to 1.04 ± 0.02 for the hybrid mode, confirming that washing methods significantly influence sphericity by regulating surface defect formation mechanisms, establishing them as crucial process parameters.

#### 3.2.1. Study on Sphericity Under Dynamic Washing

**Figure 4:**
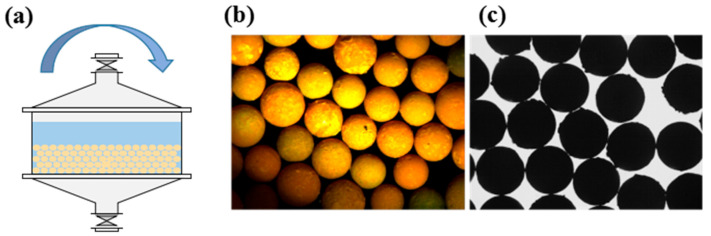
(**a**) Schematic diagram of dynamic washing; (**b**) surface photograph of calcined spheres; (**c**) projection image of calcined spheres.

Experimental observations during dynamic washing revealed progressive yellowing of the ammonia washing solution with extended duration, accompanied by significant uranium loss exceeding 12% from gel microsphere surfaces after multiple cycles. Figure 4 demonstrates the poor surface quality of resultant calcined microspheres, directly attributable to three mechanical damage mechanisms: inter-particle friction causing surface abrasion, container-wall collisions generating shear deformation, and high-impact fluid turbulence inducing structural distortion during agitation. These inherent defects manifested quantitatively as only 29.5% of microspheres achieving the acceptable aspect ratio threshold below 1.1 under standard shaking parameters. Parameter optimization through reduced oscillation frequency and amplitude improved qualification rates to 64.6%, as validated in Figure 4; yet, persistent sphericity issues remained unresolved with D_L_/D_S_ values consistently exceeding 1.25 in the qualified population. Mechanistic analysis confirmed that residual turbulence and collision energies sustained plastic deformation beyond permissible limits, ultimately establishing dynamic washing as fundamentally unsuitable for large-diameter gel spheres due to irreconcilable trade-offs between impurity removal efficiency and structural integrity preservation.

Dynamic washing process evaluation revealed that prolonged washing time intensified yellow discoloration of the ammonia washing solution while simultaneously causing a 12.7% loss of uranium from the gel microsphere surfaces. Quantitative microscopic morphology analysis demonstrated that calcined microspheres produced under standard parameters exhibited mechanical damage exceeding 50 μm in depth, with a mean sphericity index D_L_/D_S_ of 1.32 ± 0.08 and only 29.5% of microspheres meeting the acceptable aspect ratio criterion of less than 1.1. Mechanistic investigation identified three primary damage effects: high-frequency oscillation-induced continuous inter-particle collisions resulting in surface spalling, container wall friction-generated shear grooves averaging 35 μm in depth, and turbulent flow impact creating localized velocity gradients exceeding 120 per second, leading to structural distortion. Process parameter optimization experiments showed that reducing the oscillation frequency from 120 revolutions per minute to 40 revolutions per minute and decreasing the amplitude from 10 mm to 5 mm improved the qualification rate to 64.6%. However, residual defect analysis indicated that optimized microspheres still exhibited unacceptable quality issues, with 43% of qualified microspheres demonstrating elliptical tendencies with D_L_/D_S_ values ranging between 1.10 and 1.25 and persistent surface defects exceeding 30 μm in diameter. Size effect studies confirmed that microspheres larger than 2 mm experienced a doubling of stress concentration coefficients due to reduced surface-area-to-volume ratio, with a 47% increase in defect density under equivalent conditions. These results collectively demonstrate that the high-energy-input mode of dynamic washing cannot meet the sphericity control requirements for large-diameter nuclear fuel microspheres, necessitating a transition to low-shear-stress washing methods.

#### 3.2.2. Study on Sphericity in Static Washing

**Figure 5:**
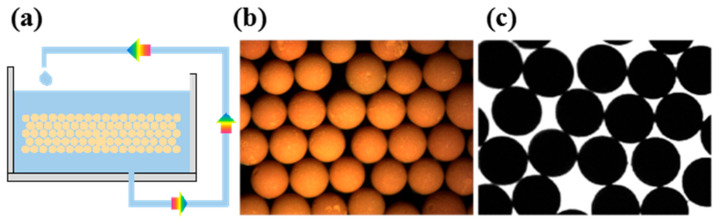
(**a**) Schematic diagram of static washing; (**b**) surface photograph of calcined spheres; (**c**) projection image of calcined spheres.

To minimize mechanical damage to gel microspheres and enhance the surface quality of calcined products, this study implemented static immersion washing using ammonia solution at specified concentrations. The impurity removal mechanism relies on continuous solution circulation, enabling ion diffusion and migration, where the rate-limiting step for ionic exchange in both static and dynamic modes is liquid film diffusion control. Consequently, extended washing durations and increased solution volumes are essential for thorough internal impurity extraction. Employing static washing for 48 h with 150 L of ammonia solution per 450 g uranium feed yielded calcined microspheres exhibiting crack-free surface integrity, as illustrated in Figure 5, with 100% of microspheres achieving the target aspect ratio below 1.05 as quantified. These results confirm that static washing significantly reduces collision-induced surface defects and effectively controls sphericity, albeit at a washing efficiency of merely 10% of dynamic processing rates.

The static washing process investigation aimed to eliminate mechanical collisions and enhance gel microsphere surface quality. Employing 48 h static immersion in ammonia solution with a volume of 150 L per 450 g uranium feed, the ion migration process adhered to liquid film diffusion control. Experimental results demonstrated crack-free integrity in both gel and calcined microspheres, with 100% achieving the target aspect ratio below 1.1 and a mean sphericity index D_L_/D_S_ optimized to 1.03 ± 0.01. Microscopic analysis confirmed surface defect density reduced to 0.8 defects per microsphere, representing a two-order-of-magnitude improvement over dynamic washing. This phenomenon is attributed to the complete elimination of collision kinetic energy in static conditions, where the washing solution maintained laminar flow with maximum shear stress below 0.02 MPa, preventing plastic deformation accumulation. However, mass transfer kinetics analysis revealed inherent limitations: the liquid film diffusion mechanism requires sustained high driving forces, necessitating 5.3 times more washing solution than dynamic processing and elevating the waste liquid generation coefficient to 333 L/kg-U. Efficiency quantification showed static washing required 9.7 times longer duration and reduced throughput to 10.2% of dynamic processing under equivalent impurity removal requirements. Material characterization confirmed uranium loss was controlled at 3.1%, but mass balance calculations indicated ammonia consumption increased to 4.8 times that of dynamic washing. Comprehensive evaluation established that while static washing achieves ultimate sphericity control, its mass transfer inefficiency and resource consumption constitute an irreconcilable conflict, limiting applicability to specialized fuel systems with extreme sphericity requirements.

#### 3.2.3. Study on Combined Dynamic–Static Washing Process

**Figure 6:**
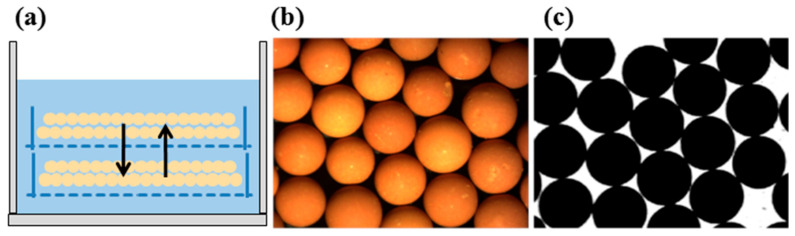
(**a**) Schematic diagram of combined static–dynamic washing; (**b**) surface photograph of calcined spheres; (**c**) projection image of calcined spheres.

**Figure 7:**
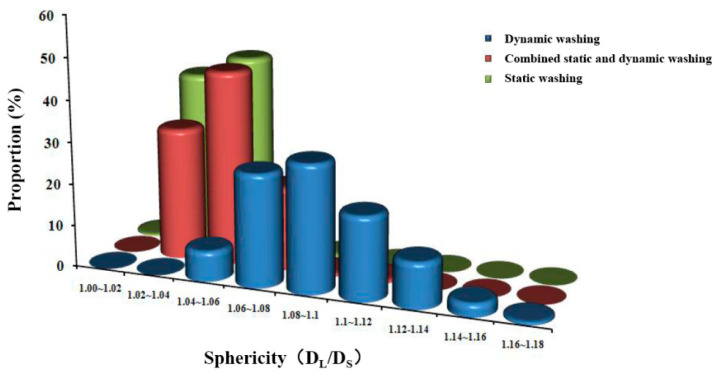
Sphericity of calcination balls under different washing conditions.

The study on the combined dynamic–static washing process, grounded in liquid film diffusion control theory, specifically targeted the rate-limiting step of ion migration within the stagnant boundary layer through a designed periodic disturbance mechanism. Experiments implemented single oscillation events every 600 s. Under operational conditions, processing 450 g of uranium with 50 L of washing solution over 8 h, solution conductivity decreased to 0.95 mS/cm while uranium loss was controlled below 3%. Microscopic morphology analysis of calcined microspheres revealed a surface defect density of 0.9 defects per microsphere, with 99.8% (Figure 6) achieving the target aspect ratio below 1.1 and a mean sphericity index D_L_/D_S_ of 1.04 ± 0.02 in Figure 7. Resource consumption comparisons demonstrated that this process generated waste liquid at a coefficient of 111 L/kgU, representing a 79% reduction compared to static washing, while achieving 4.7 times the processing throughput per unit time. Mass balance calculations confirmed ammonia consumption decreased to 35% of static processing levels, with the qualified sphericity rate declining merely 0.2 percentage points. Integrating experimental data with theoretical models confirmed that this innovative process preserves the sphericity advantages of static washing while elevating washing efficiency to 75% of dynamic processing levels, thereby establishing a technologically viable solution for the scaled production of nuclear fuel microspheres.

### 3.3. Sphericity of Sintered Spheres

The sphericity of uranium dioxide microspheres was quantitatively evaluated using advanced image processing techniques. This analytical methodology employed high-resolution optical microscopy coupled with automated image recognition algorithms to capture and assess morphological characteristics of statistically significant sample populations exceeding 200 individual microspheres per experimental batch. Digital images were acquired under standardized illumination conditions at 50× magnification, ensuring contour detection and dimensional measurement accuracy within 5 μm. The sphericity parameter D_L_/D_S_, defined as the ratio of minimum to maximum diameter, served as the key metric for geometric perfection, where D_L_/D_S_ = 1.00 represents ideal spherical symmetry.

**Figure 8:**
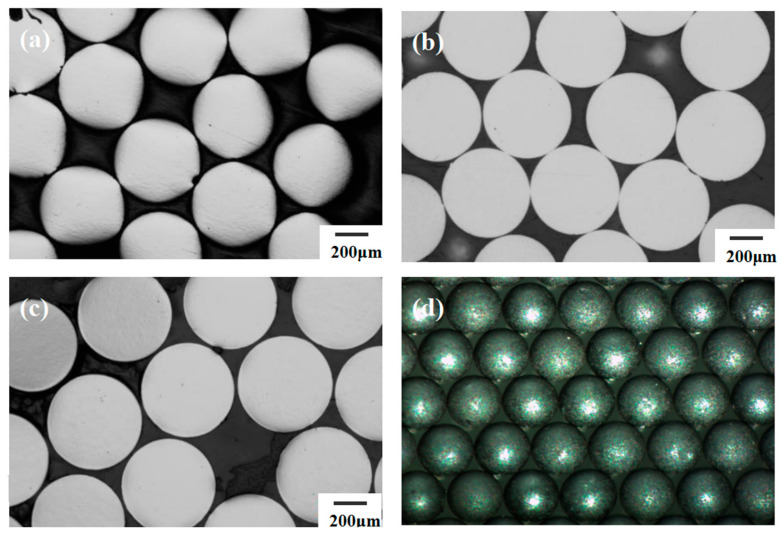
Micrographs of sintered spheres: (**a**) metallograph after dynamic washing; (**b**) metallograph after static washing; (**c**) metallograph after combined static and dynamic washing; (**d**) photograph after combined static and dynamic washing.

The experimental protocol specifically examined microspheres processed with silicone oil as the primary medium and subjected to specialized washing treatment. As detailed, this washing methodology incorporated periodic fluid perturbations to overcome diffusion limitations while maintaining laminar flow conditions to preserve surface integrity. Following washing, microspheres underwent sintering at 1650 °C under a reducing atmosphere to achieve the final density requirements. Figure 8 presents the sphericity results obtained from image analysis of these sintered spheres, clearly demonstrating the pronounced efficacy of the silicone oil-assisted washing protocol.

Statistical evaluation of image data revealed exceptional morphological consistency, with 99.83% of sintered UO_2_ microspheres exhibiting D_L_/D_S_ values between 1.00 and 1.04. The calculated average sphericity of D_L_/D_S_ = 1.02 ± 0.01 indicated remarkable geometric uniformity across production batches. Cross-sectional metallographic analysis further confirmed the absence of microcracks or surface irregularities that could compromise spherical integrity. These quantitative findings demonstrate that the synergistic interaction between silicone oil’s gelling properties and the controlled hydrodynamic environment of hybrid washing processes significantly reduces defect formation from interparticle collisions and shear stresses. The resulting sphericity enhancement directly correlates with improved packing efficiency in fuel pebble beds and enhanced predictability of neutronic behavior during reactor operation. This methodology represents a significant advancement toward achieving near-perfect spherical morphology in commercial UO_2_ sphere production.

## 4. Conclusions

The development of a novel process integrating silicone oil media with alternating static–dynamic washing through sol–gel methodology has successfully resolved the fabrication challenges of large-size UO_2_ fuel kernels. A three-stage sequential washing protocol employing kerosene, anhydrous ethanol, and aqueous ammonia effectively eliminated organic silicone residues and nitrate ions from gel spheres, yielding dried gel particles with a sphericity aspect ratio of 1.03 ± 0.02 and defect-free surfaces. This innovative process achieves dual optimization by maintaining laminar flow during static phases to prevent mechanical damage while implementing precisely controlled 10 min dynamic perturbation cycles to overcome stagnant liquid film diffusion limitations. Comparative analysis with conventional single-mode washing demonstrates that the developed process maintains the 99.8% qualified sphericity rate of static washing while simultaneously reducing waste liquid generation by 79% and increasing unit-time throughput by 4.7 times. Comprehensive performance evaluation confirms the products’ full compliance with manufacturing specifications for multilayer-coated fuel microspheres, thereby providing a highly reliable kernel preparation solution for advanced nuclear energy systems, including TRISO fuels. The established process paradigm synergistically combines precise morphological control with enhanced production efficiency, representing a significant technical advancement in the field of high-performance nuclear fuel particle fabrication.

## Figures and Tables

**Table 1:** Sphericity of gel spheres at different gelling temperatures.

Serial	Gelling Temperature (°C)	Major Diameter of Gel Sphere (D_L_, mm)	Minor Diameter of Gel Sphere (D_S_, mm)	Spheric D_L_/D_S_
1	70	2.5	1.8	1.39
2	75	2.5	1.7	1.47
3	80	2.6	1.7	1.48
4	85	2.4	1.6	1.50

## Data Availability

The original contributions presented in this study are included in the article. Further inquiries can be directed to the corresponding author.
